# Genome-wide association study identifies genetic susceptibility loci and pathways of radiation-induced acute oral mucositis

**DOI:** 10.1186/s12967-020-02390-0

**Published:** 2020-06-05

**Authors:** Da-Wei Yang, Tong-Min Wang, Jiang-Bo Zhang, Xi-Zhao Li, Yong-Qiao He, Ruowen Xiao, Wen-Qiong Xue, Xiao-Hui Zheng, Pei-Fen Zhang, Shao-Dan Zhang, Ye-Zhu Hu, Guo-Ping Shen, Mingyuan Chen, Ying Sun, Wei-Hua Jia

**Affiliations:** 1 State Key Laboratory of Oncology in South China, Collaborative Innovation Center for Cancer Medicine, Guangdong Key Laboratory of Nasopharyngeal Carcinoma Diagnosis and Therapy, Sun Yat-sen University Cancer Center, Guangzhou, People’s Republic of China; 2 School of Public Health, Sun Yat-sen University, Guangzhou, People’s Republic of China; 3 Department of Radiation Oncology, Sun Yat-sen University First Affiliated Hospital, Guangzhou, People’s Republic of China; 4 Department of Nasopharyngeal Carcinoma, Sun Yat-sen University Cancer Center, Guangzhou, People’s Republic of China; 5 Department of Radiation Oncology, Sun Yat-sen University Cancer Center, Guangzhou, People’s Republic of China; 6 Department of Radiation Oncology, Affiliated Cancer Hospital & Institute of Guangzhou Medical University, Guangzhou, People’s Republic of China

**Keywords:** Radiation injuries, Oral mucositis, Radiogenomics, Genome-wide association study, Functional mapping

## Abstract

**Background:**

Radiation-induced oral mucositis (OM) is one of the most common acute complications for head and neck cancer. Severe OM is associated with radiation treatment breaks, which harms successful tumor management. Radiogenomics studies have indicated that genetic variants are associated with adverse effects of radiotherapy.

**Methods:**

A large-scale genome-wide scan was performed in 1467 nasopharyngeal carcinoma patients, including 753 treated with 2D-CRT from Genetic Architecture of the Radiotherapy Toxicity and Prognosis (GARTP) cohort and 714 treated with IMRT (192 from the GARTP and 522 newly recruited). Subgroup analysis by radiotherapy technique was further performed in the top associations. We also performed physical and regulatory mapping of the risk loci and gene set enrichment analysis of the candidate target genes.

**Results:**

We identified 50 associated genomic loci and 64 genes via positional mapping, expression quantitative trait locus (eQTL) mapping, chromatin interaction mapping and gene-based analysis, and 36 of these loci were replicated in subgroup analysis. Interestingly, one of the top loci located in TNKS, a gene relevant to radiation toxicity, was associated with increased OM risk with OR = 3.72 of the lead SNP rs117157809 (95% CI 2.10–6.57; *P *= 6.33 × 10^−6^). Gene set analyses showed that the 64 candidate target genes were enriched in the biological processes of regulating telomere capping and maintenance and telomerase activity (Top *P* = 7.73 × 10^−7^).

**Conclusions:**

These results enhance the biological understanding of radiotherapy toxicity. The association signals enriched in telomere function regulation implicate the potential underlying mechanism and warrant further functional investigation and potential individual radiotherapy applications.

## Background

Radiotherapy is the primary treatment regimens for head and neck cancer. Oral mucositis (OM) is one of the most common acute radiation-induced toxicities of head and neck cancer, which contributes to difficult eating and drinking, weight loss, fatigue, pain, sleep deprivation, and functional impairment [1]. Intolerable oral mucositis could cause unplanned treatment breaks, negatively affecting the efficacy of treatment regimens and treatment outcomes [2]. Due to the high tumor control of nasopharyngeal carcinoma (NPC) by radiotherapy, more attentions has been paid to the adverse effects of radiotherapy, especially radiation-induced OM [3].

In a large proportion of patients, the use of opioid analgesics does not adequately palliate symptoms. Therefore, symptomatic management of mucositis is insufficient to avoid negative clinical outcomes, and there is a clear need for agents that reduce the incidence of mucositis [1]. Radiogenomics studies have suggested that common genetic variants are associated with radiotherapy adverse effects, and a single-nucleotide polymorphism-based predictive assay along with clinical factors could be used to estimate the risk of a patient with cancer developing adverse effects from radiotherapy. Such an assay could be used for personalized therapy and for the prevention of severe adverse effects, which could improve quality of life for patients [4].

For adverse reactions to radiotherapy, tailoring treatment dose by genetic risk is considered to achieve individualized treatment. It proposed a hypothesis that germline genetics contribute to the development of radiation injury. So far, the mechanisms of radiation-induced normal tissue toxicity are complex and are not fully understood. However, it has been reported that there are at least 14 canonical pathways taking part in the development of OM in patients treated with radiochemotherapy [5]. Our aim is to identify new loci and pathways associated with the development of radiation-induced OM through a genome-wide association approach in a population from southern China.

## Materials and methods

### Study objects

The participants were recruited from two sections. 960 subjects were screened between 2005 and 2007 from the GARTP study (Genetic Architecture of the Radiotherapy Toxicity and Prognosis, registered with http://www.chictr.org.cn/, ChiCTR-ROC-17012658), according to the following criteria: pathologically confirmed NPC, previously untreated, no previous radiotherapy and/or chemotherapy, receiving the whole course of radical radiotherapy, and adult (age older than 18). For the patients who were treated by 2D-CRT, the accumulated radiation doses to the primary tumor were 68–76Gy with two Gy per fraction. For the patients treated by IMRT, the prescribed treatment protocol was 68–70Gy for 30–33 fractions to the planning target volume (PTV) of gross tumor volume of the primary (GTV-P) and 64–68Gy for 30–33 fractions to the PTV of nodal gross tumor volume (GTV-N). We recruited additional 553 NPC patients from the Sun Yat-sen University Cancer Center (SYSUCC) of China in 2006–2014. One patient was excluded because he did not complete the whole course of radiotherapy. The characteristics of patients, including age, gender, TNM stage (using 2009 7th UICC/AJCC staging system), radiation technique and treatment scheme were recorded.

### Mucositis evaluation

Oral mucositis caused by radiotherapy was observed and recorded. It was evaluated and classified as grade 0–5 based on the acute radiation toxicity grading criterion of the Radiation Therapy Oncology Group or European Organization for Research and Treatment of Cancer (RTOG/EORTC) [6]. According to the grading results, we divided the patients into two groups: severe OM (grade ≥ 3) and mild OM (grade ≤ 2).

### Genotyping, quality control and imputation

Genomic DNA was extracted from whole peripheral blood samples using a commercial DNA extraction kit (Qiagen) and was quantified using PicoGreen reagent (Invitrogen). We genotyped GARTP study samples on the Human610-Quad Chip and others on Infinium Global Screening Array-24 BeadChip. Genotyping and quality control for the Human610-Quad chip can be found in our previous publication [7]. For the Infinium Global Screening Array-24, we generated a cluster file using our in-house data including about 2000 samples from our cancer center, and called genotypes according to the manufacture’s protocol [8, 9]. The variants with low call rates, poor clustering metrics or extreme heterozygosity rate were manually re-clustered or removed. We then performed quality control at sample level and at SNP level according to the following criteria: (1) individuals level: call rate < 95%, gender discrepancies, heterozygosity rate outliers (> 6 sd.), unexpected duplicates or probable relatives based on pairwise identity by descent (PI_HAT > 0.5), and population stratification outliers (> 6 sd.); (2) SNPs level: non-autosomal chromosomes, call rate < 95%, minor allele frequencies (MAF) < 0.001, and deviated from Hardy–Weinberg equilibrium (HWE) (*P *< 10^−12^). All filtered samples were imputed by a two-stage imputation approach, using SHAPEIT2 [10] for phasing and IMPUTE2 [11] for imputation. The imputation was performed in 5-Mb nonoverlapping intervals. SNPs with a frequency > 1% and that were imputable with INFO > 0.8 were included in the downstream analysis.

We then merged overlapping SNPs and conducted further quality control to the SNPs. We excluded SNPs with call rates < 95%, deviated from Hardy–Weinberg equilibrium (*P *< 10^−12^), or MAF < 0.01. We performed quality control filtering using PLINK 1.09 [12]. Finally, a total of 1467 patients (945 patients from the GARTP study and 522 patients recruited in 2006–2014) and 3,968,928 genetic variants were analyzed in GWAS.

### Genome-wide association analyses

Univariate logistic regression analyses were performed by comparison of clinical factors with OM. Considering the collinearity among the clinical variables, multivariate regression analysis was further performed with the filtered variables. The significantly associated clinical factors in multivariate regression were adjusted in genome-wide association analyses. Genome-wide association analyses were performed under additive genetic effects assumption, using a logistic regression model adjusting treatment scheme, radiation technology and the first five eigenvectors of principal components as covariates. We also created quantile–quantile plot and Manhattan plot using the R package “qqman”. A quantile–quantile plot was used to evaluate the overall significance of the GWAS, and the deviation of the observed versus the expected distribution of the *P* values was represented by the inflation factor (λ_GC_). Considering the different incidence rates in the different radiation technology subgroups (2D-CRT and IMRT), we performed further association analysis using logistic models, only adjusting for the treatment scheme in the two subgroups, respectively to examine the top variants.

### Genomic risk loci and functional annotation

Functional annotation was performed with FUMA [13], an online platform for the functional mapping of genetic variants. We first defined ‘independent significant SNPs’ as those surpassing a predefined threshold *P* value (1 × 10^−4^) and showing moderate to low linkage disequilibrium (r^2^ < 0.6). We further defined ‘lead SNPs’ as the subset of independent SNPs (r^2^ < 0.1). Additionally, we defined genomic risk loci by merging LD blocks of independent significant SNPs that have close physical position (< 250 kb). All known SNPs in the 1000 genome data that have (r^2^ > 0.6) with any of the independent significant SNP were included for annotation, and the region containing all of these ‘candidate SNPs’ was considered to be a single independent genomic locus. All LD information was calculated from 1000G phase3 East Asian population [14].

Functional consequences for the SNPs were obtained by performing ANNOVAR [15] gene-based annotation using Ensembl genes. SNPs were matched according to chromosome, position, reference, and alternative alleles, and were annotated by CADD scores (scores > 12.37 indicate deleterious SNP [16]), RegulomeDB scores [17] (lower scores indicate higher potentiality of regulatory function), and by chromatin states predicted by hidden Markov model based on 5 chromatin marks for 127 epigenomes in the Roadmap Epigenomics Project (lower scores ≤ 7 represent higher accessibility of the genomic regions). CADD scores integrate diverse annotations into a single measure that correlates with pathogenicity, disease severity, experimentally measured regulatory effects and complex trait associations.

### Gene mapping

SNPs in genomic risk loci were mapped to genes in FUMA using three strategies.

First, position mapping was based on the physical distances (within a 10-kb window) from known protein-coding genes in the human reference assembly (GRCh37 or hg19). The second strategy, eQTL mapping, used information from three data repositories (GTEx [18], Blood eQTL browser [19], and BIOS QTL browser [20]) and mapped SNPs to genes based on a significant eQTL association (i.e., where the expression of the gene is associated with allelic variation at the SNP). eQTL mapping was based on cis-eQTLs (local regulatory effect within 1 Mb). A false discovery rate (FDR) of 0.05 was applied to define significant eQTL association. The third strategy, chromatin interaction mapping, mapped SNPs to the promoter regions of genes based on significant chromatin interactions. This type of mapping was a 3D DNA–DNA interaction between the SNP region and a gene region, without a distance boundary. FUMA currently contains Hi-C data for 21 tissue/cell types from the study [21]. Because chromatin interactions are often defined in a certain resolution (40 kb), an interaction region may span multiple genes. Hence, this method would map all SNPs within these regions to genes in the corresponding interaction region. To prioritize candidate genes, we integrated predicted enhancers and promoters in certain tissue and cell types from the Roadmap Epigenomics Project [22], including blood, gastrointestinal tissue and skin. Using the information, FUMA selected chromatin interactions for which one region involved in the interaction overlapped with predicted enhancers and the other overlapped with predicted promoters 250 bp upstream and 500 bp downstream of the TSS of a gene. We used an FDR of 1 × 10^−6^ to define significant interactions.

### Gene set analysis

Genes implicated by mapping of GWAS SNPs were further investigated using the GENE2FUNC procedure in FUMA, which provides hypergeometric tests of enrichment of the list of mapped genes in MSigDB gene sets [23], including BioCarta, KEGG, Reactome, and Gene Oncology (GO). The adjusted *P* value (FDR) for gene set enrichment analysis was supplied by the Benjamini–Hochberg method. The threshold of adjusted *P*-value was 0.05. The minimum number of input genes overlapping with a tested gene set to be reported as significant was two.

## Results

### Population characteristics

The study population was composed of 1467 NPC. Of those, 349 patients (23.79%) developed severe OM (grade ≥ 3) after radiotherapy, and no grade 5 mucositis was observed. The clinical characteristics were analyzed by univariate logistic regression. Tumor stage, clinical stage, radiation technique and treatment scheme were reported to be associated with severe OM (Table 1). The severe OM incidence rates were significantly different for patients who received different radiation therapies, with rates of 14.9% and 33.2% for patients treated with 2D-CRT and IMRT, respectively. The clinical characteristics of different subgroups (2D-CRT and IMRT) are shown in Additional file 1: Table S1. Compared with radiotherapy alone, patients treated with induction chemotherapy and/or adjuvant chemotherapy showed similar OM risk with an OR of 1.07 (95% CI 0.55–2.09). However, patients treated with concurrent chemoradiotherapy had a higher risk of severe OM with an OR of 6.96 (95% CI 4.50–10.77) compared to patients treated with radiotherapy alone. The results of multivariate logistic regression indicated that different radiation techniques and treatment schemes were significant clinical factors for the incidence of radiation-induced OM (Additional file 2: Table S2) and were considered as covariates in the GWAS.

**Table 1:** Association of clinical factors and the risk of acute oral mucositis

Characteristics	Oral mucositis		*P*	OR	95% CI
	Grade ≥ 3	Grade ≤ 2			
Number of patients	349 (23.79%)	1118 (76.21%)			
Age (Mean ± SD)	45.12 ± 10.71	45.18 ± 10.94	0.934	1.00	0.99–1.01
Gender			0.524		
Male	258	807		–	–
Female	91	311		0.92	0.69–1.20
Clinical stage			0.047		
I–II	71	286		–	–
III–IV	278	832		1.35	1.00–1.81
Tumor stage^a^			0.035		
1–2	85	338		–	–
3–4	264	780		1.35	1.02–1.77
Radiation technique			< 0.001		
2D-CRT	112	641		–	–
IMRT	237	477		2.84	2.21–3.67
Treatment scheme			< 0.001		
RT alone	24	325		–	–
RT + IC/AC	15	190	0.845	1.07	0.55–2.09
CCRT	310	603	< 0.001	6.96	4.50–10.77

95% CI, 95% confidence interval; SD, standard deviation; 2D-CRT, two-dimensional conventional radiotherapy; IMRT, intensity modulated radiation therapy; RT, radiotherapy; RT + IC/AC, Radiotherapy with induction chemotherapy and/or adjuvant chemotherapy; CCRT, Concurrent chemoradiotherapy

^a^The patients were staged according to the 2009 7th UICC/AJCC staging system

### SNPs associated with severe oral mucositis

**Fig. 1:**
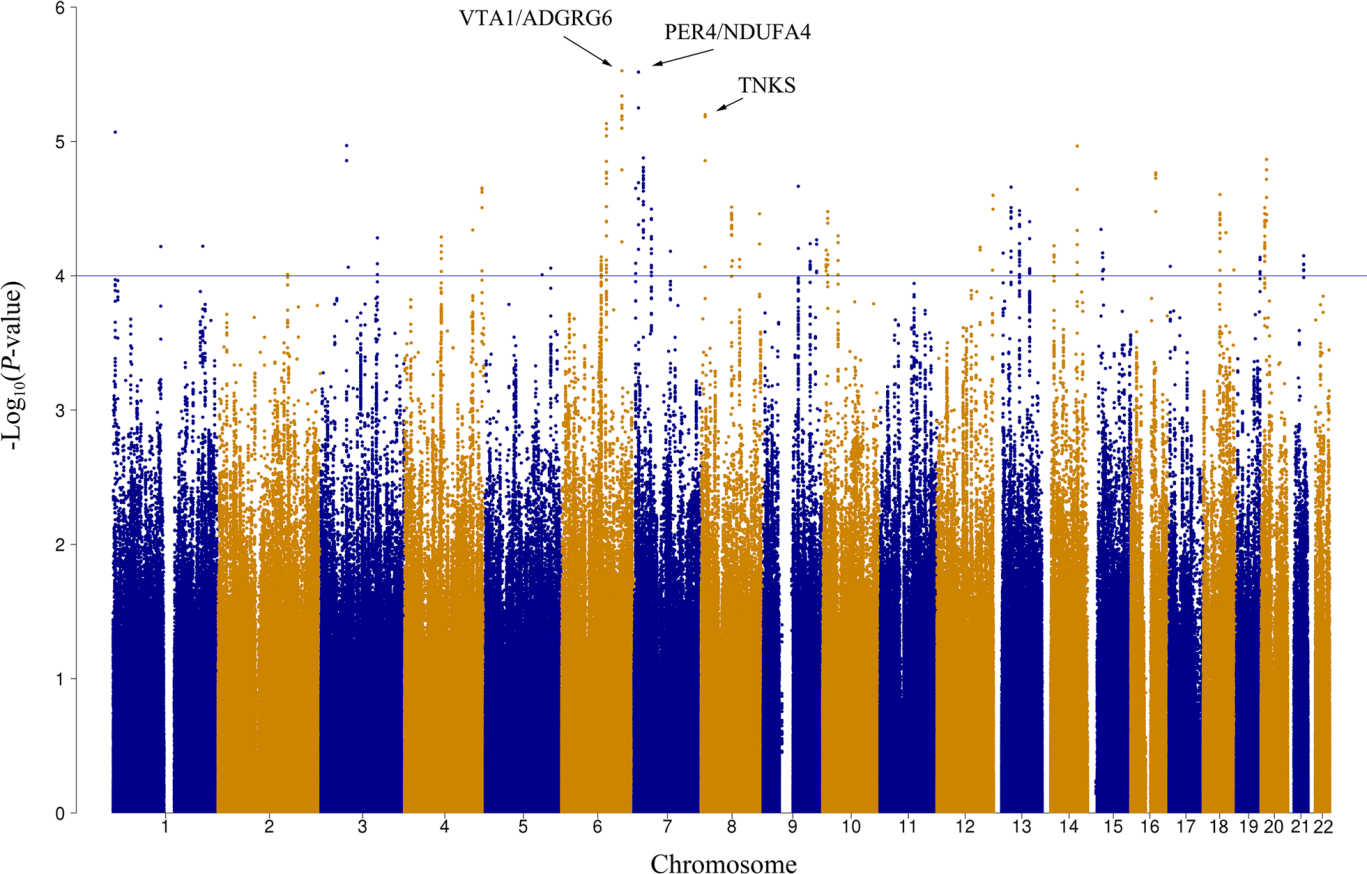
Manhattan plot of the genome-wide *P*-values of associations. Associations were assessed using logistic regression analysis with adjustment for treatment, the radiation technique and the top five principal components of population stratification. The blue line indicates the threshold for suggestive significance: *P *< 1 × 10^−4^

A total of 3,968,928 SNPs were included in the genome-wide association analysis under an additive assumption using a logistic regression model, adjusting radiation technique and treatment scheme. The distribution of the observed versus the expected *P* values are shown in the quantile–quantile plot with λ_GC_ = 1.01 (Additional file 3: Figure S1). The three top lead SNPs were rs9484606 in the intergenic region of chromosome 6 (OR = 1.70, 95% CI 1.36–2.13, *P *= 2.98 × 10^−6^, VTA1/ADGRG6), rs16876733 in the intergenic region of chromosome 7 (OR = 1.95, 95% CI 1.47–2.59, *P *= 3.05 × 10^−6^, PER4/NDUFA4), and rs117157809 in the intron region of TNKS (OR = 3.72, 95% CI 2.10–6.57, *P *= 6.33 × 10^−6^), respectively (Fig. 1). We selected the independent SNPs with *P*-value < 1×10^−4^ in GWAS analysis using all patients, and further examined them in the two treatment subgroups (2D-CRT and IMRT) under the threshold of *P *= 0.05. The resulting significant SNPs are shown in Table 2.

**Table 2:** Association results of SNPs in the GWAS with all patients, and subgroup analysis

SNP	Locus	CHR	MA	GENE	All patients			
					N	OR (95% CI)	P	MAF (case/control)
rs9484606	15	6	C	VTA1/ADGRG6	1454	1.70 (1.36–2.13)	2.98 × 10^−6^	0.25/0.18
rs16876733	17	7	C	PER4/NDUFA4	1452	1.95 (1.47–2.59)	3.05 × 10^−6^	0.16/0.09
rs117157809	21	8	C	TNKS	1423	3.72 (2.10–6.57)	6.33 × 10^−6^	0.05/0.01
rs4433399	1	1	A	MIR4417/MIR4689	1449	1.69 (1.34–2.12)	8.53 × 10^−6^	0.23/0.16
rs3094972	18	7	T	STEAP1B	1460	0.46 (0.32–0.65)	1.33 × 10^−5^	0.07/0.12
rs11908263	49	20	A	BTBD3/LOC101929486	1450	2.06 (1.49–2.86)	1.36 × 10^−5^	0.11/0.07
rs1562525	5	3	A	FHIT	1467	1.75 (1.36–2.25)	1.39 × 10^−5^	0.18/0.12
rs9941163	43	16	T	GOT2/APOOP5	1447	2.56 (1.67–3.93)	1.72 × 10^−5^	0.07/0.03
rs59936027	35	13	A	WBP4/MIR3168	1439	1.61 (1.29–2.00)	2.19 × 10^−5^	0.26/0.18
rs7673990	10	4	G	TENM3	1455	1.81 (1.38–2.38)	2.22 × 10^−5^	0.15/0.09
rs13227327	16	7	A	SDK1	1417	1.62(1.30-2.03)	2.24 × 10^−5^	0.27/0.19
rs561697	44	18	G	LINC00907	1420	1.71 (1.33–2.19)	2.49 × 10^−5^	0.20/0.14
rs10957542	22	8	T	EYA1	1433	0.42 (0.28–0.63)	3.08 × 10^−5^	0.05/0.09
rs6133617	48	20	T	PLCB1	1456	1.49 (1.23–1.79)	3.11 × 10^−5^	0.55/0.46
rs1079866	19	7	G	LINC01449/INHBA	1465	1.59 (1.28–1.98)	3.19 × 10^−5^	0.26/0.19
rs9570470	36	13	A	LINC00378/MIR3169	1461	0.50 (0.36–0.70)	3.28 × 10^−5^	0.07/0.13
rs7068532	29	10	A	DHTKD1	1446	1.60(1.28-2.00)	3.33 × 10^−5^	0.26/0.19
rs4909632	24	8	G	KHDRBS3/LOC101927915	1447	1.49 (1.24–1.80)	3.46 × 10^−5^	0.56/0.48
rs12430962	37	13	A	SNORA107/LINC00375	1461	0.64 (0.52–0.79)	3.96 × 10^−5^	0.22/0.30
rs138519422	40	15	C	OTUD7A/CHRNA7	1415	3.14 (1.81–5.43)	4.52 × 10^−5^	0.05/0.02
rs6814005	8	4	T	MAPK10	1461	2.34 (1.55–3.53)	5.16 × 10^−5^	0.07/0.03
rs80231193	27	9	C	LHX2/NEK6	1421	2.79 (1.70–4.60)	5.41 × 10^−5^	0.05/0.02
rs10816756	26	9	C	IKBKAP	1464	1.87 (1.38–2.53)	5.77 × 10^−5^	0.12/0.07
rs7147736	38	14	A	LINC00645/FOXG1-AS1	1420	1.77 (1.34–2.33)	5.99 × 10^−5^	0.16/0.11
rs79945158	2	1	A	LOC100996251	1423	3.39 (1.87–6.16)	6.06 × 10^−5^	0.04/0.02
rs79549170	31	12	G	GNPTAB/DRAM1	1439	1.98 (1.42–2.77)	6.13 × 10^−5^	0.10/0.06
rs17143701	28	10	C	LINC00708/LOC105755953	1463	1.86 (1.37–2.51)	6.46 × 10^−5^	0.12/0.07
rs79052434	41	15	G	DPH6-AS1	1459	0.50 (0.36–0.70)	6.76 × 10^−5^	0.07/0.12
rs2850108	50	21	A	CLDN14	1452	1.48 (1.22–1.79)	7.11 × 10^−5^	0.43/0.34
rs7742301	13	6	T	CASC6/EPHA7	1467	1.62 (1.28–2.05)	7.25 × 10^−5^	0.21/0.15
rs16987032	47	19	A	GALP	1467	1.66 (1.29–2.13)	7.59 × 10^−5^	0.18/0.12
rs28375758	12	5	C	KIF4B/SGCD	1456	1.59 (1.26–2.01)	8.78 × 10^−5^	0.24/0.18
rs36058653	46	18	G	ZNF407/ZADH2	1437	1.65 (1.28–2.11)	9.06 × 10^−5^	0.19/0.13
rs76156855	32	12	T	GPR133	1444	3.69 (1.92–7.10)	9.10 × 10^−5^	0.03/0.01
rs9752986	4	2	T	KCNH7/FIGN	1433	2.07 (1.43–2.98)	9.79 × 10^−5^	0.09/0.05
rs10050575	11	5	A	C5orf66	1421	3.48 (1.86–6.53)	9.85 × 10^−5^	0.04/0.01

SNP	IMRT^a^				2D-CRT^b^			
	N	OR (95% CI)	P	MAF (case/control)	N	OR (95% CI)	P	MAF (case/control)
rs9484606	707	1.38 (1.20–1.59)	2.70 × 10^−2^	0.22/0.17	747	2.45 (2.03–2.96)	2.14 × 10^−6^	0.31/0.19
rs16876733	699	2.02 (1.69–2.42)	6.56 × 10^−5^	0.17/0.10	753	1.79 (1.39–2.30)	2.10 × 10^−2^	0.13/0.09
rs117157809	704	3.34 (2.31–4.84)	1.10 × 10^−3^	0.04/0.01	719	5.06 (3.13–8.18)	6.99 × 10^−4^	0.05/0.02
rs4433399	696	1.74 (1.50–2.02)	2.73 × 10^−4^	0.22/0.14	753	1.64 (1.36–1.98)	1.01 × 10^−2^	0.25/0.17
rs3094972	708	0.42 (0.33–0.53)	1.06 × 10^−4^	0.06/0.13	752	0.49 (0.36–0.66)	1.75 × 10^−2^	0.07/0.10
rs11908263	707	1.88 (1.51–2.34)	3.89 × 10^−3^	0.10/0.06	743	2.27 (1.75–2.94)	1.65 × 10^−3^	0.15/0.07
rs1562525	714	1.92 (1.62–2.28)	9.15 × 10^−5^	0.18/0.11	753	1.51 (1.22–1.86)	4.92 × 10^−2^	0.18/0.13
rs9941163	709	2.58 (1.95–3.41)	6.85 × 10^−4^	0.07/0.03	738	2.08 (1.45–2.98)	4.14 × 10^−2^	0.06/0.03
rs59936027	709	1.49 (1.30–1.71)	4.41 × 10^−3^	0.26/0.20	730	1.85 (1.53–2.24)	1.30 × 10^−3^	0.26/0.18
rs7673990	710	1.64 (1.37–1.96)	5.96 × 10^−3^	0.13/0.09	745	2.02 (1.60–2.54)	1.87 × 10^−3^	0.17/0.09
rs13227327	682	1.44 (1.25–1.66)	1.10 × 10^−2^	0.27/0.21	735	2.04 (1.69–2.47)	1.87 × 10^−4^	0.29/0.18
rs561697	682	1.69 (1.44–1.98)	8.13 × 10^−4^	0.20/0.13	738	1.77 (1.42–2.21)	8.31 × 10^−3^	0.21/0.14
rs10957542	693	0.44 (0.34–0.56)	1.10 × 10^−3^	0.05/0.10	740	0.39 (0.27–0.56)	8.98 × 10^−3^	0.04/0.09
rs6133617	708	1.53 (1.36–1.73)	4.10 × 10^−4^	0.54/0.45	748	1.45 (1.24–1.70)	1.97 × 10^−2^	0.56/0.46
rs1079866	714	1.68 (1.46–1.93)	2.26 × 10^−4^	0.27/0.18	751	1.54 (1.27–1.86)	2.28 × 10^−2^	0.26/0.20
rs9570470	708	0.50 (0.41–0.61)	7.73 × 10^−4^	0.07/0.13	753	0.51 (0.38–0.68)	1.95 × 10^−2^	0.07/0.14
rs7068532	702	1.66 (1.44–1.91)	3.47 × 10^−4^	0.26/0.18	744	1.47 (1.22–1.78)	4.34 × 10^−2^	0.24/0.20
rs4909632	706	1.40 (1.24–1.58)	6.09 × 10^−3^	0.53/0.46	741	1.67 (1.42–1.96)	1.80 × 10^−3^	0.62/0.50
rs12430962	708	0.66 (0.58–0.75)	2.09 × 10^−3^	0.23/0.31	753	0.60 (0.50–0.73)	8.88 × 10^−3^	0.21/0.29
rs138519422	697	3.38 (2.33–4.89)	1.14 × 10^−3^	0.05/0.01	718	2.65 (1.69–4.16)	2.87 × 10^−2^	0.05/0.02
rs6814005	711	2.27 (1.75–2.94)	1.54 × 10^−3^	0.08/0.03	750	2.49 (1.72–3.60)	1.40 × 10^−2^	0.06/0.03
rs80231193	705	3.01 (2.14–4.23)	1.05 × 10^−3^	0.05/0.02	716	2.98 (1.98–4.49)	7.81 × 10^−3^	0.06/0.03
rs10816756	711	1.80 (1.49–2.18)	1.97 × 10^−3^	0.12/0.07	753	2.12 (1.62–2.78)	6.30 × 10^−3^	0.11/0.07
rs7147736	683	1.82 (1.52–2.18)	9.13 × 10^−4^	0.16/0.10	737	1.67 (1.31–2.12)	2.92 × 10^−2^	0.15/0.12
rs79945158	688	2.59 (1.75–3.83)	1.57 × 10^−2^	0.03/0.02	735	5.92 (3.66–9.57)	2.29 × 10^−4^	0.05/0.02
rs79549170	697	1.67 (1.34–2.08)	2.05 × 10^−2^	0.09/0.06	742	2.64 (2.02–3.46)	3.58 × 10^−4^	0.12/0.06
rs17143701	713	1.97 (1.61–2.41)	7.27 × 10^−4^	0.12/0.07	750	1.74 (1.36–2.23)	2.81 × 10^−2^	0.13/0.08
rs79052434	711	0.51 (0.41–0.64)	1.72 × 10^−3^	0.07/0.12	748	0.51 (0.38–0.68)	2.05 × 10^−2^	0.07/0.13
rs2850108	700	1.38 (1.22–1.56)	9.09 × 10^−3^	0.39/0.32	752	1.64 (1.40–1.92)	2.55 × 10^−3^	0.49/0.36
rs7742301	714	1.54 (1.33–1.79)	4.29 × 10^−3^	0.20/0.15	753	1.64 (1.34–2.00)	1.47 × 10^−2^	0.24/0.14
rs16987032	714	1.37 (1.17–1.61)	4.69 × 10^−2^	0.17/0.14	753	2.41 (1.95–2.97)	4.19 × 10^−5^	0.21/0.11
rs28375758	704	1.37 (1.18–1.59)	3.72 × 10^−2^	0.22/0.19	752	2.07 (1.69–2.53)	2.22 × 10^−4^	0.27/0.18
rs36058653	702	1.54 (1.31–1.81)	6.13 × 10^−3^	0.18/0.13	735	2.09 (1.68–2.60)	8.54 × 10^−4^	0.20/0.13
rs76156855	704	4.29 (2.82–6.53)	4.66 × 10^−4^	0.04/0.01	740	3.91 (2.06–7.42)	3.24 × 10^−2^	0.02/0.01
rs9752986	688	1.93 (1.52–2.45)	5.92 × 10^−3^	0.09/0.04	745	2.27 (1.68–3.06)	6.91 × 10^−3^	0.10/0.05
rs10050575	688	2.61 (1.75–3.89)	1.67 × 10^−2^	0.03/0.01	733	5.79 (3.48–9.64)	5.37 × 10^−4^	0.04/0.01

Case, RTOG grade ≥ 3; Control, RTOG grade ≤ 2

SNP, single nucleotide polymorphism; CHR, chromosome; MA, minor allele; MAF, minor allele frequency; OR, odds ratio for minor allele; 95% CI, 95% confidence interval

^a^The subgroup of patients who received intensity modulated radiation therapy

^b^The subgroup of patients who received two-dimensional conventional radiotherapy

### Gene mapping

Using three gene mapping strategies (position mapping, eQTL mapping and chromatin interaction mapping) in FUMA, we further mapped the significant association variants to genes and identified 50 genomic risk loci and 64 mapped genes associated with radiation-induced oral mucositis (Additional files 4, 5: Table S3, S4). The results of the overlapped SNPs and genes in the subgroup analysis are shown in Table 2.

**Fig. 2:**
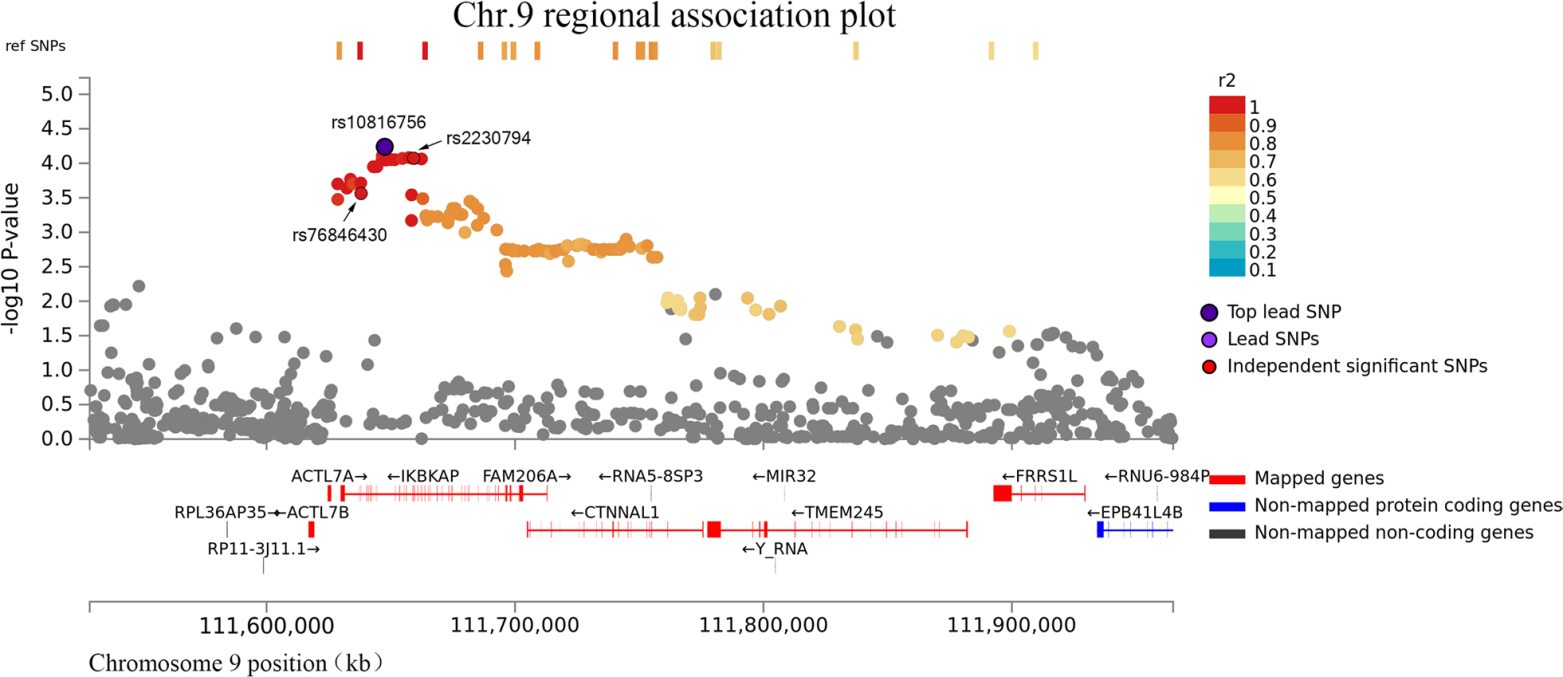
Regional plot of the association of rs10810756. The -log_10_*P*-value (y-axis) of SNPs are presented according to their chromosomal positions (x-axis). The lead SNP (labeled by rs ID) is indicated by a deep purple circle, and the r^2^ values of the rest of the SNPs with the top genotyped SNP are indicated by different colors. SNPs that are not in LD with any of the independent significant SNPs (with r^2^ ≤ 0.4) are gray

**Fig. 3:**
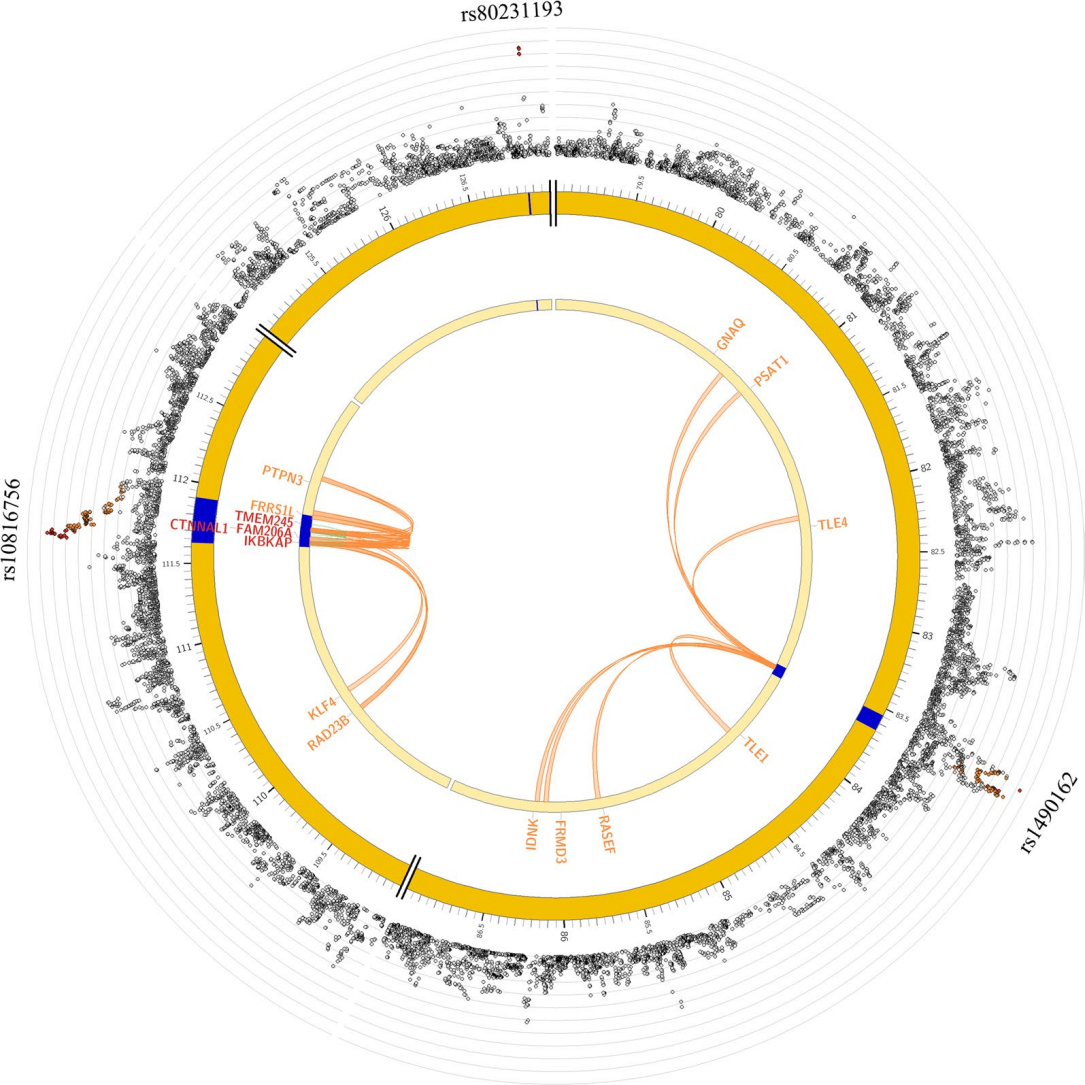
Cross-locus interactions for genomic regions associated with radiation-induced oral mucositis. Circos plots showing genes on chromosome 9 that were implicated through the genomic risk loci (blue areas) by positional mapping, by chromatin interaction mapping (orange font), eQTL mapping (green font), or by both chromatin interaction and eQTL mapping (red font). The outer layer shows a Manhattan plot containing the -log_10_ P-value of each SNP in the GWAS analysis of radiation-induced oral mucositis (n = 1467 individuals)

The two genes IKBKAP and DHTKD1 were mapped by all three strategies. IKBKAP was located at the chromosome 9 locus, and its lead SNP rs10816756 was located in the intron of the gene with an OR of 1.87 for the minor allele (95% CI 1.38–2.53, *P* = 5.77 × 10^−5^). Two SNPs rs2230794 and rs76846430, located at the exon of IKBKAP, were both in complete LD with rs10816756 (r^2^ = 1, Fig. 2). rs2230794 is a missense variant (OR = 1.83, 95% CI 1.35–2.49, *P* = 9.85 × 10^−5^), and rs76846430 is a splice-site variant (OR = 1.78, 95% CI 1.30–2.42, *P *= 2.7 × 10^−4^). We further performed expression quantitative trait locus (eQTL) analysis and found that with the increasing number of risk alleles of rs10816756, there was a higher mRNA level of IKBKAP in the whole peripheral blood (Additional file 6: Table S5). We further analyzed chromatin functional interaction in the risk loci, and 8 genes were identified to interact with the chromatin at that site, such as IKBKAP, KLF4, and RAD23B (Fig. 3 and Additional file 7: Table S6). The other gene, DHTKD1, was located at the chromosome 10 locus, and its lead SNP rs7068532 was in the intron region with an OR of the minor allele of 1.60 (95% CI 1.28–2.00, *P *= 3.33 × 10^−5^). eQTL analysis indicated that the whole peripheral blood mRNA level of DHTKD1 decreased when the risk alleles increased (Additional file 6: Table S5). The risk locus had a chromatin interaction with the gene DHTKD1 (Additional file 8: Figure S2 and Additional files 5, 6: Tables S4, S5).

In addition, we searched the 64 mapped genes in the GeneRIF dataset to examine their relevance to radiation-induced OM. There are two low frequency variants, rs117157809 and rs6814005, which were located in the introns of TNKS and MAPK10, respectively. Patients carrying the minor alleles of rs117157809 tended to have higher risks of developing severe OM with a per allele OR of 3.72 (95% CI 2.10–6.57, *P* = 6.33 × 10^−6^). The association was consistent with an OR of 3.34 (95% CI 2.31–4.84, *P* = 1.10 × 10^−3^) in patients treated by IMRT and with an OR of 5.06 (95% CI 3.13–8.18, *P* = 6.99 × 10^−4^) in patients treated by 2D-CRT. The SNP rs79488099 located in the 3′-UTR of TNKS was in modest LD with the lead SNP rs117157809 (rs79488099: r^2^ = 0.63, *P *= 1.48 × 10^−4^), and the CADD score of rs79488099 was 16.17 indicating a deleterious mutation. A similar result for rs6814005 is shown in Table 2. Another lead SNP, rs13227327, was located in the intron of SDK1 with an OR of the minor allele of 1.62 (95% CI 1.30–2.03, *P* = 2.24 × 10^−5^). In that risk loci, two SNPs, rs601424 and rs671694, were both located in the exons of SDK1 and were in modest LD with rs13227327 (rs601424: r^2^ = 0.69, *P* = 4.54 × 10^−4^; rs671694: r^2^ = 0.69, *P *= 3.31 × 10^−4^; Additional file 9: Figure S3).

### Gene-set based analysis

The FUMA tool implicated 64 genes providing more extensive information on the likely consequences of relevant genetic variants. Gene-set based analysis was performed using these genes to further evaluate the underlying disease mechanisms responsible for the genetic signals. The 20 significant GO biological processes are listed in Additional file 10: Table S7. Among those gene sets, there were 8 GO gene sets involved in the regulation of telomere or telomerase activity, including in the regulation of telomere capping (*P* = 7.73 × 10^−7^), the positive regulation of telomerase activity (*P* = 2.13 × 10^−6^), and the positive regulation of telomere maintenance (*P* = 1.77 × 10^−5^). Four genes (TNKS, NEK2, NBN and KLF4) recurred in these GO sets. In addition, 4 of 20 significant gene sets were involved in DNA metabolism, biosynthesis and replication. One of those pathways was the Wnt signaling pathway, which was reported to regulate radioresistance [24].

## Discussion

In the present study, we found that some clinical factors were associated with radiation-induced OM, especially chemotherapy concurrent with radiotherapy, which is promoted for cancer control and is widely used for the cancer treatment [25]. However, it greatly increases the radiation sensitivity of normal tissue and the occurrence of radiation-induced OM. Adjusting these clinical factors, our genome-wide association study identified 50 risk loci and 64 mapped genes by using a total sample size of nearly 1500. Many of the OM-associated genes are involved in telomere biological processes, including telomere capping, maintenance and telomerase activity, while some other genes participate in DNA biological processes, including DNA metabolism, biosynthesis and replication.

To our knowledge, this study is the largest GWAS for oral mucositis in NPC patients treated with radiotherapy. Published studies have investigated the associations between SNPs and radiation-induced OM in head and neck cancer and in nasopharyngeal carcinoma. Most of them evaluated the associations by adopting the strategy of candidate genes. Those candidate genes and pathways include DNA damage and repair involved in double-strand breaks repair genes [26] and the base excision repair pathway [27]. Other important cellular signaling pathways include the Wnt/β-catenin pathway [28], cell cycle regulated genes, the NF-κB pathways [29], angiogenesis-related genes [30], and GAS5 lncRNAs [31]. Those studies were based on small sample sizes of 100–500 and evaluated limited number of SNPs in the candidate genes, and the significant SNPs were found at the level of an uncorrected *P* value of 0.05. Only one study showed genome-wide level analysis in a total of 24 patients with NPC [32]. For the studies of other radiation-induced side effects, many of the susceptibility genes identified were implicated in DNA damage response and repair pathways, oxidative stress and apoptosis [33]. Our gene sets-based analysis identified the Wnt signaling pathway, which has been reported to be related with radiation-induced OM [28]. More importantly, we identified other potential pathways, including telomere and DNA biological processes. In particular, the gene set of telomere biological process has a significant impact on the radiosensitivity of patients.

It has been demonstrated that telomere dysfunction is correlated with delayed DNA break repair kinetics and with sensitivity to ionizing radiation. For example, the telomerase-deficient mouse models demonstrated that short telomeres determined a condition of hypersensitivity to ionizing radiation, and consequently, had a decreased survival rate [34]. In vitro experiments have suggested that irradiation sensitivity of non-transformed human epithelial cells is augmented with telomere dysfunction because short dysfunctional telomeres interfered with efficient DNA repair by joining radiation-induced DNA broken ends, and it also reduced the repair fidelity of DNA broken-ends [35]. Furthermore, the study has formulated that the importance of telomeres in predicting individual radiosensitivity of cancer patients [36].

In this study, the gene sets related with telomere function such as telomere capping, maintenance and telomerase activity were mainly implicated by TNKS, NEK2, KLF4 and NBN. These genes were reported to be linked to the radiation-induced damage. For example, depletion of TNKS is associated with a defective damage response observed by degraded proteasome-mediated DNA-PKcs, including increased sensitivity to ionizing radiation-induced mutagenesis, chromosome aberration (terminal deletion), telomere fusion, and cell killing [37]. The activity of NEK2 was reported to be inhibited by ionizing radiation, and this response was dependent on ATM and on PP1 binding to NEK2 [38]. The absence of NEK2 promoted apoptosis and reduced cell numbers [39]. Other evidence indicated that NEK2 promoted glioma stem cell radioresistance through the regulation of EZH2 [40]. In addition, KLF4 was reported to prevent centrosome amplification and to exhibit antiapoptotic activity following γ-radiation-induced DNA damage [41, 42]. It has been further confirmed in animal experiments that KLF4 was a radio-protective factor for the intestine following γ-radiation-induced gut injury in mice [43]. NBN has been reported to have an association with radiation-induced oral mucositis [44], and its mutation was found in the radiosensitivity-related syndrome and Nijmegen breakage syndrome [45]. In vitro studies have demonstrated that the influence of NBN on radiation hypersensitivity was accompanied by enhanced γ-radiation-induced apoptosis in human lymphoblastoid cells [46]. Our finding further elucidated the underlying mechanisms of radiation-induced damage.

Some genes that are significant in both of the treatment subgroups are of further interest, including IKBKAP, SDK1 and MAPK10. IKBKAP is located on chromosome 9 in risk locus 26. rs10816756 is the lead SNP of that locus, having a modest sign of *P *= 5.77 × 10^−5^. IKBKAP was identified as a scaffold protein that plays a role in the regulation of activation of the mammalian stress response via the c-Jun N-terminal kinase (JNK)-signaling pathway [47], and JNK signaling pathway has been suggested to be involved in radiation-induced OM pathobiology [48]. SDK1, an adhesion molecule, is activated by cellular stress especially in conditions with the reactive oxygen species [49]. In addition, the gene MAPK10, a member of the MAPK signaling pathway, is capable of regulation p38-MAPK, PI3K-MAPK and other cascades, which are involved in the response to ionizing radiation [50]. A preliminary analysis performed in lymphocytes from three radiation-exposed individuals showed that MAPK10 was an induced gene associated with cell responses to ionizing radiation [51].

## Conclusions

In summary, our genome-wide association study identified 50 genomic risk loci and 64 candidate genes for radiation-induced oral mucositis. The combined strategies of functional annotation and gene mapping using biological data resources provided extensive information on the likely consequences of relevant genetic variants. We highlight several genes implicated through multiple routes, and we put forward a rich set of plausible gene targets and biological mechanisms for functional follow-up. Gene set analyses contributed novel insight into the underlying telomere pathways, confirming the importance of telomere function in developing radiation-induced adverse effects. Larger samples and functional experiments are required. The current study provides new leads and functional hypotheses for radiation-induced oral mucositis, and it is important for predicting individual radiosensitivity and for promoting personalized radiotherapy strategies.

## Supplementary information

**Additional file 1: Table S1.** Clinical characteristics of two subgroups with radiation technology.

**Additional file 2: Table S2.** Multivariate logistic regression analysis of clinical factors and acute oral mucositis.

**Additional file 3: Figure S1.** Quantile–quantile plot of genome-wide *P* value of associations.

**Additional file 4: Table S3.** Association *P*-values for all lead SNPs for severe oral mucositis.

**Additional file 5: Table S4.** All genes mapped in SNP-based (FUMA) for severe oral mucositis.

**Additional file 6: Table S5.** eQTL linking GWAS risk SNPs of severe oral mucositis to mapped genes.

**Additional file 7: Table S6.** Chromatin interaction regions linking GWAS risk loci of severe oral mucositis to mapped genes.

**Additional file 8: Figure S2.** Cross-locus interactions for genomic regions in chromosome 10 associated with radiation-induced oral mucositis.

**Additional file 9: Figure S3.** Regional plots of association for rs13227327.

**Additional file 10: Table S7.** The top 20 significantly gene sets analysis for radiation-induced oral mucositis.

## Data Availability

The datasets used and analysed during the current study are available from the corresponding author on reasonable request.

## Abbreviations

OM: Oral mucositis
GARTP cohort: Genetic Architecture of the Radiotherapy Toxicity and Prognosis cohort
eQTL: Expression quantitative trait locus
NPC: Nasopharyngeal carcinoma
RTOG/EORTC: Radiation Therapy Oncology Group or European Organization for Research and Treatment of Cancer
PTV: Planning target volume
SYSUCC: Sun Yat-sen University Cancer Center
MAF: Minor allele frequency
GWAS: Genome-wide association study
