# Supplementary material for: Genome-wide association study identifies genetic susceptibility loci and pathways of radiation-induced acute oral mucositis
Source: J Transl Med. 2020 Jun 5;18:224. doi: 10.1186/s12967-020-02390-0 (PMC7275566; doi:10.1186/s12967-020-02390-0)
Supplement: Supplementary file 1 — Additional file 1: Table S1. Clinical characteristics of two subgroups with radiation technology. [file 12967_2020_2390_MOESM1_ESM.docx]

**Additional Table S1. Clinical characteristics of two subgroups with radiation technology**

| **Characteristics** | **2D-CRT** | | **IMRT** | |
| --- | --- | --- | --- | --- |
|  | **Grade≥3** | **Grade≤2** | **Grade≥3** | **Grade≤2** |
| Number of patients | 112(15%) | 641(85%) | 237(33%) | 477(67%) |
| Age（Mean±SD） | 46.87±10.65 | 46.23±10.72 | 44.30±10.66 | 43.77±11.11 |
| Sex |  |  |  |  |
| Male | 74(66%) | 454(71%) | 184(78%) | 353(74%) |
| Female | 38(34%) | 187(29%) | 53(22%) | 124(26%) |
| Clinical stage† |  |  |  |  |
| Ⅰ-Ⅱ | 30(27%) | 190(30%) | 41(17%) | 96(20%) |
| Ⅲ-Ⅳ | 82(73%) | 451(70%) | 196(83%) | 381(80%) |
| Tumor stage |  |  |  |  |
| 1-2 | 34(30%) | 208(32%) | 51(22%) | 130(27%) |
| 3-4 | 78(70%) | 433(68%) | 186(78%) | 347(73%) |
| Treatment scheme |  |  |  |  |
| RT alone | 11(10%) | 254(40%) | 13(5%) | 71(15%) |
| RT+IC/AC | 9(8%) | 177(27%) | 6(3%) | 13(3%) |
| CCRT | 92(82%) | 210(33%) | 218(92%) | 393(82%) |

Abbreviations: 95% CI, 95% confidence interval; SD, standard deviation; 2D-CRT, two-dimensional conventional radiotherapy; IMRT, Intensity Modulated Radiation Therapy; RT, Radiotherapy; RT+IC/AC, Radiotherapy with induction chemotherapy and/or adjuvant chemotherapy; CCRT, Concurrent chemoradiotherapy.

† The patients were staged according to the 2009 7th UICC/AJCC stageing system.
