# Supplementary material for: Genome-wide association study identifies genetic susceptibility loci and pathways of radiation-induced acute oral mucositis
Source: J Transl Med. 2020 Jun 5;18:224. doi: 10.1186/s12967-020-02390-0 (PMC7275566; doi:10.1186/s12967-020-02390-0)
Supplement: Supplementary file 2 — Additional file 2: Table S2. Multivariate logistic regression analysis of clinical factors and acute oral mucositis. [file 12967_2020_2390_MOESM2_ESM.docx]

**Additional Table S2: Multivariate logistic regression analysis of clinical factors and acute oral mucositis**

|  | *β* | SE | *P* | OR | 95%CI |
| --- | --- | --- | --- | --- | --- |
| Treatment scheme |  |  | <0.001 |  |  |
| RT alone | — | — | — | 1(reference) | — |
| RT+IC/AC | 0.14 | 0.34 | 0.678 | 1.15 | 0.59-2.26 |
| CCRT | 1.76 | 0.23 | <0.001 | 5.80 | 3.69-9.09 |
| Radiation technique |  |  |  |  |  |
| 2D-CRT | — | — | — | 1(reference) | — |
| IMRT | 0.45 | 0.14 | 0.002 | 1.57 | 1.18-2.08 |

Abbreviations: SE, standard error; 2D-CRT, two-dimensional conventional radiotherapy; IMRT, Intensity Modulated Radiation Therapy; RT, Radiotherapy; RT+IC/AC, Radiotherapy with induction chemotherapy and/or adjuvant chemotherapy; CCRT, Concurrent chemoradiotherapy.
