# Supplementary material for: Genome-wide association study identifies genetic susceptibility loci and pathways of radiation-induced acute oral mucositis
Source: J Transl Med. 2020 Jun 5;18:224. doi: 10.1186/s12967-020-02390-0 (PMC7275566; doi:10.1186/s12967-020-02390-0)
Supplement: Supplementary file 3 — Additional file 3: Figure S1. Quantile–quantile plot of genome-wide P value of associations. [file 12967_2020_2390_MOESM3_ESM.pptx]

## Slide 1
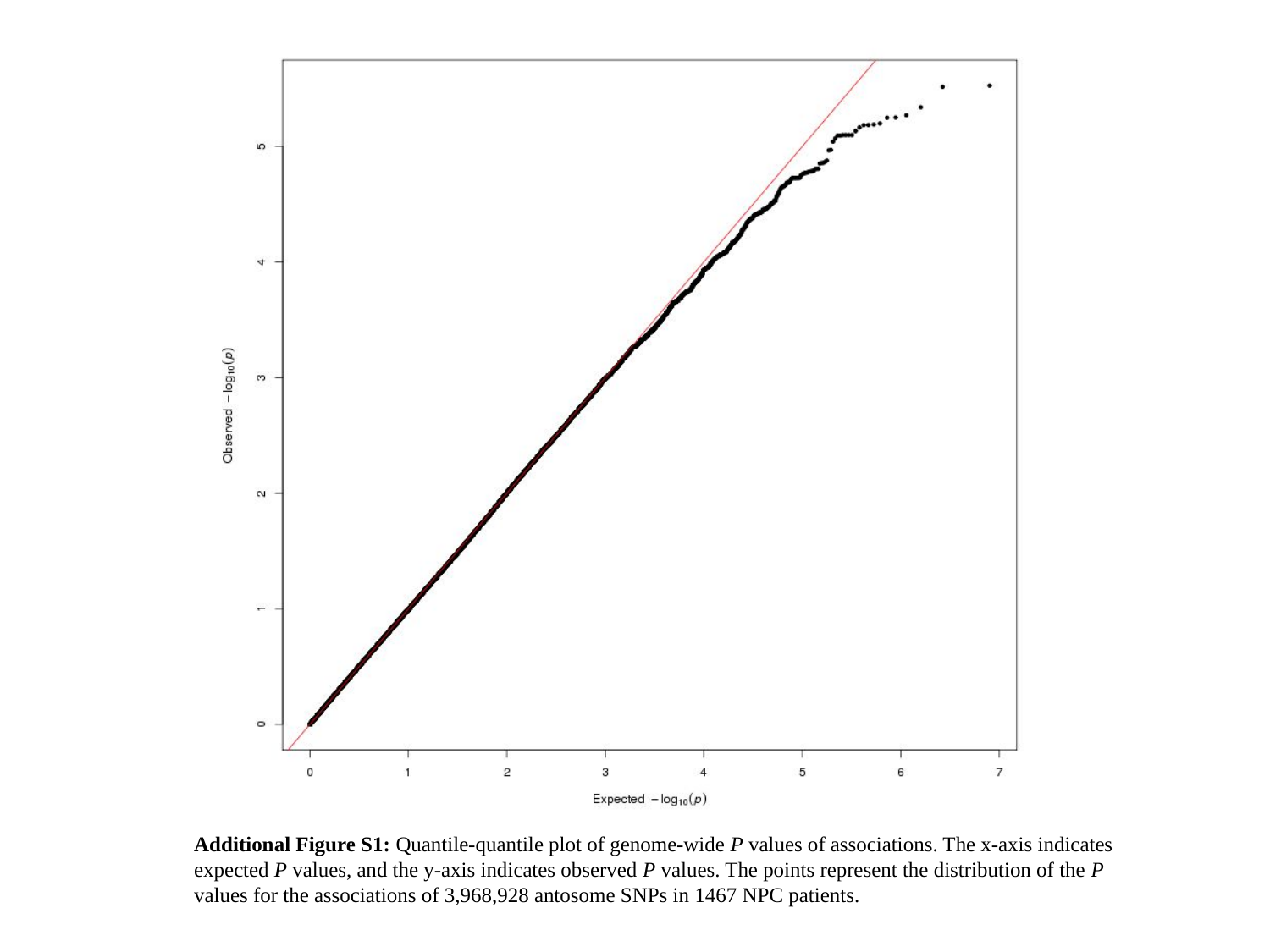

Additional Figure S1: Quantile-quantile plot of genome-wide P values of associations. The x-axis indicates expected P values, and the y-axis indicates observed P values. The points represent the distribution of the P values for the associations of 3,968,928 antosome SNPs in 1467 NPC patients.
