# Supplementary material for: Genome-wide association study identifies genetic susceptibility loci and pathways of radiation-induced acute oral mucositis
Source: J Transl Med. 2020 Jun 5;18:224. doi: 10.1186/s12967-020-02390-0 (PMC7275566; doi:10.1186/s12967-020-02390-0)
Supplement: Supplementary file 8 — Additional file 8: Figure S2. Cross-locus interactions for genomic regions in chromosome 10 associated with radiation-induced oral mucositis. [file 12967_2020_2390_MOESM8_ESM.pptx]

## Slide 1
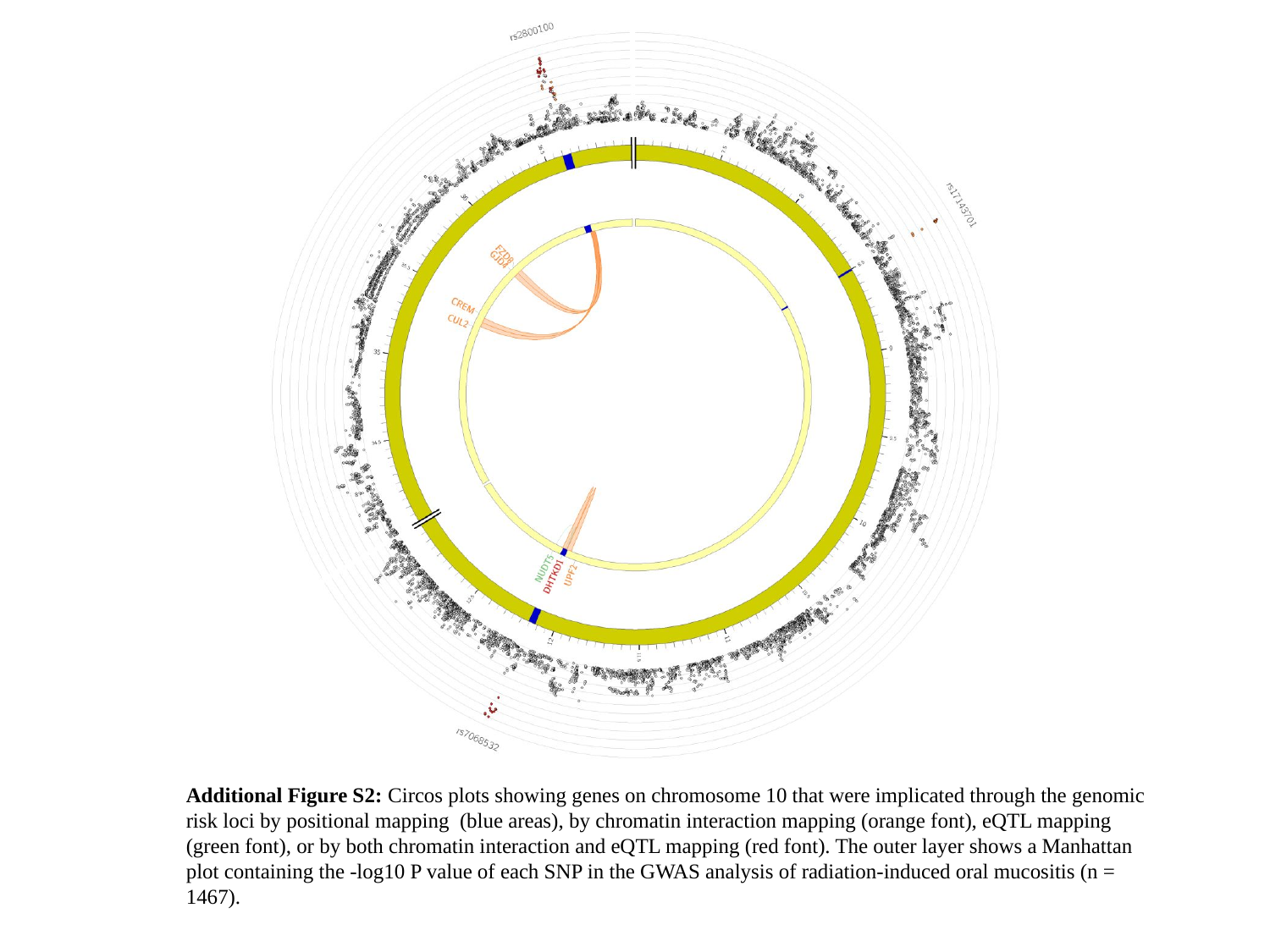

Additional Figure S2: Circos plots showing genes on chromosome 10 that were implicated through the genomic risk loci by positional mapping (blue areas), by chromatin interaction mapping (orange font), eQTL mapping (green font), or by both chromatin interaction and eQTL mapping (red font). The outer layer shows a Manhattan plot containing the -log10 P value of each SNP in the GWAS analysis of radiation-induced oral mucositis (n = 1467).
