# Supplementary material for: Genome-wide association study identifies genetic susceptibility loci and pathways of radiation-induced acute oral mucositis
Source: J Transl Med. 2020 Jun 5;18:224. doi: 10.1186/s12967-020-02390-0 (PMC7275566; doi:10.1186/s12967-020-02390-0)
Supplement: Supplementary file 9 — Additional file 9: Figure S3. Regional plots of association for rs13227327. [file 12967_2020_2390_MOESM9_ESM.pptx]

## Slide 1
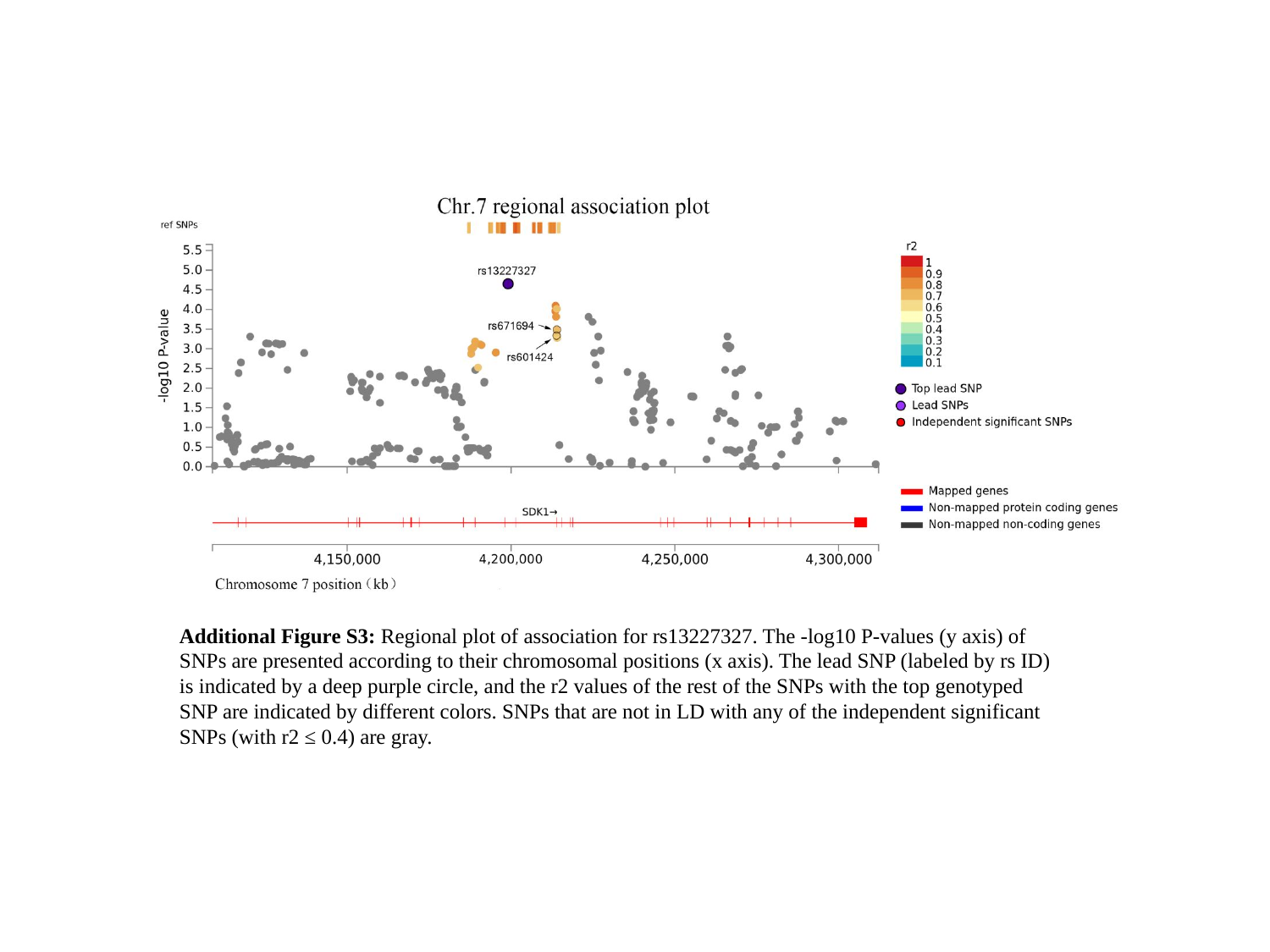

Additional Figure S3: Regional plot of association for rs13227327. The -log10 P-values (y axis) of SNPs are presented according to their chromosomal positions (x axis). The lead SNP (labeled by rs ID) is indicated by a deep purple circle, and the r2 values of the rest of the SNPs with the top genotyped SNP are indicated by different colors. SNPs that are not in LD with any of the independent significant SNPs (with r2 ≤ 0.4) are gray.
